# Infection Prophylaxis in Urological Diagnostic and Surgical Procedures – A narrative review and Recommendations

**DOI:** 10.1590/S1677-5538.IBJU.2025.0333

**Published:** 2025-07-20

**Authors:** José Carlos Truzzi, André Avarese Figueiredo, João Antônio Pereira Correia, José Pontes, Marcelo Cabrini, Lorenzo S. Ramacciotti, Caio Cesar Citatini de Campos, Karin Marise Jaeger Anzolch, Antônio Peixoto de Lucena Cunha, Bruno V. Mestrinho, Leandro Koifman, Marcos Broglio, Ubirajara Barroso, Luciano A. Favorito, José de Bessa

**Affiliations:** 1 Universidade Federal de São Paulo – Unifesp Escola Paulista de Medicina São Paulo SP Brasil Escola Paulista de Medicina, Universidade Federal de São Paulo – Unifesp, São Paulo, SP, Brasil;; 2 Universidade Federal de Juiz de Fora Faculdade de Medicina Juiz de Fora MG Brasil Faculdade de Medicina, Universidade Federal de Juiz de Fora – UFJF, Juiz de Fora, MG, Brasil;; 3 Hospital Federal da Lagoa Serviço de Urologia Rio de Janeiro RJ Brasil Serviço de Urologia, Hospital Federal da Lagoa, Rio de Janeiro, RJ, Brasil;; 4 Hospital das Clínicas Instituto do Câncer do Estado de São Paulo São Paulo SP Brasil Instituto do Câncer do Estado de São Paulo, Hospital das Clínicas - FMUSP, São Paulo, SP, Brasil;; 5 University of Southern California USC Institute of Urology Los Angeles United States of América USC Institute of Urology, University of Southern California, Los Angeles, United States of América;; 6 Hospital do Servidor Público Municipal de São Paulo Serviço de Urologia São Paulo SP Brasil Serviço de Urologia, Hospital do Servidor Público Municipal de São Paulo, São Paulo, SP, Brasil;; 7 Hospital Moinhos de Vento Serviço de Urologia Porto Alegre RS Brasil Serviço de Urologia, Hospital Moinhos de Vento, Porto Alegre, RS, Brasil;; 8 Faculdade de Ciências Médicas de Minas Gerais Departamento de Urologia Belo Horizonte MG Brasil Departamento de Urologia, Faculdade de Ciências Médicas de Minas Gerais, Belo Horizonte, MG, Brasil;; 9 Universidade Rio Verde – Campus Luziânia Departamento de Urologia Luziânia GO Brasil Departamento de Urologia, Universidade Rio Verde – Campus Luziânia, Luziânia, GO, Brasil;; 10 Hospital Municipal Souza Aguiar Serviço de Urologia Rio de Janeiro RJ Brasil Serviço de Urologia, Hospital Municipal Souza Aguiar, Rio de Janeiro, RJ, Brasil;; 11 Faculdade de Ciências Médicas da Santa Casa de São Paulo São Paulo SP Brasil Faculdade de Ciências Médicas da Santa Casa de São Paulo, São Paulo, SP, Brasil;; 12 Universidade Federal da Bahia Faculdade de Medicina Salvador BA Brasil Faculdade de Medicina, Universidade Federal da Bahia – UFBA, Salvador, BA, Brasil;; 13 Universidade do Estado do Rio de Janeiro Unidade de Pesquisa Urogenital Rio de Janeiro RJ Brasil Unidade de Pesquisa Urogenital, Universidade do Estado do Rio de Janeiro – UERJ, Rio de Janeiro, RJ, Brasil;; 14 Universidade Estadual de Feira de Santana Departamento de Cirurgia Feira de Santana BA Brasil Departamento de Cirurgia, Universidade Estadual de Feira de Santana – UEFS, Feira de Santana, BA, Brasil;

**Keywords:** Surgical Wound Infection, Prophylactic Surgical Procedures, Review [Publication Type]

## Abstract

**Objectives:**

In this review we will provide recommendations for surgical site infection (SSI) prophylaxis in urological diagnostic and surgical procedures.

**Material and Methods:**

We performed a narrative review of the literature in PubMed (Medline), EMBASE, LILACS, Web of Science, and Cochrane Collaboration databases using the terms "infection," "surgery," "urology," and "antibiotic prophylaxis"

**Results:**

We suggest recommendations of prophylactic antibiotic in the follow procedures: prostate biopsy, Urethrocystoscopy, Extracorporeal Shock Wave Lithotripsy (ESWL) for Urinary Stones, endoscopic ureterolithotripsy, percutaneous nephrolitothomy, Transurethral Resection of the Prostate (TURP) and Prostatic Enucleation, Transurethral Resection of Bladder Tumor (TURBT), Intravesical Botulinum Toxin Injection, surgical correction of female stress incontinence, Surgical Correction of Pelvic Organ Prolapse, urological prosthesis implantation and Open, Laparoscopic, and Robotic Urologic Surgery

**Conclusions:**

Consideration in SSI prophylaxis and the prophylactic antibiotic regimens in several urological procedures are show in this paper and will be useful to urologic practice.

## INTRODUCTION

Surgical site infection (SSI) affects the incision, organ, or space involved in an operative procedure. The incidence ranges from 0.5% to 15% in surgical patients and can reach up to 38% in some studies ( 1 - 3 ). Several factors influence the risk of SSI, including the degree of procedure contamination, preoperative hair removal, operative time, patient comorbidities, overall health status, and proper antibiotic prophylaxis. Implementing evidence-based preventive measures can prevent approximately half of SSIs ( 1 , 4 ).

General preventive measures include stringent operating room hygiene, instrument sterilization, restriction of unnecessary personnel movement in the operating room, and proper surgical attire, which are beyond the scope of this manuscript. SSI prophylaxis specific to patient care includes both non-antibiotic and antibiotic measures. While antibiotics are crucial in reducing surgical infections, non-antibiotic measures—many historically adopted in surgical practice before being validated by clinical trials—remain equally essential.

A critical consideration in SSI prophylaxis is the rising rate of bacterial resistance to antibiotics, now recognized as a public health concern. This has prompted the World Health Organization (WHO) to advocate for urgent implementation of antimicrobial stewardship programs to optimize antibiotic use.

This document aims to provide recommendations for SSI prophylaxis in urological diagnostic and surgical procedures.

## MATERIALS AND METHODS

Key topics were determined through a consensus meeting among the authors to develop the recommendations, followed by a literature review. PubMed (Medline), EMBASE, LILACS, Web of Science, and Cochrane Collaboration databases were searched using the terms "infection," "surgery," "urology," and "antibiotic prophylaxis." Articles published exclusively in English from 2012–2024 were initially screened by title and abstract, and selected for full-text screening if they met inclusion criteria defined by the purpose of the review. Additional relevant publications were identified through reference list screening of the selected studies. Studies published before 2012 were included if they contained relevant concepts or data not covered by more recent studies.

The recommendations were developed following the *Grading of Recommendations Assessment, Development, and Evaluation* (GRADE) system, by the Ministry of Health's guidelines. Under the GRADE framework, the level of evidence is classified as high, moderate, low, or very low, while the strength of the recommendation is categorized as strong or weak. Each recommendation is accompanied by its corresponding GRADE assessment, presented as (GRADE: level of evidence, strength of recommendation) ( 5 ). In cases where the authors disagreed about the strength of a recommendation, the majority consensus was adopted. The lowest grade was chosen when the literature provided varying grades of recommendation for different aspects of a procedure.

## RESULTS

### General Aspects of Surgical Infection Prophylaxis

#### Non-Antibiotic Measures for Surgical Infection Prophylaxis

**Glycemic Control:** Preoperative glycemic control is one of the most critical parameters for reducing the risk of SSIs. The U.S. Centers for Disease Control and Prevention (CDC) recommends maintaining blood glucose levels below 200 mg/dL in both diabetic and non-diabetic patients ( 1 , 6 , 7 ). A meta-analysis proposed a stricter threshold of 150 mg/dL for both groups, although this approach carries a higher risk of hypoglycemia ( 8 ). Compared to conventional glycemic control protocols, intensive perioperative insulin regimens have been shown to reduce SSI incidence in cardiac and abdominal surgeries ( 7 ).

**Preoperative Bathing:** Among antiseptic practices aimed at reducing infection risk, bathing with soap and water effectively removes transient microbiota and inactivates skin flora, lowering the risk of SSI by threefold ( 9 ). However, the effectiveness of a 4% chlorhexidine gluconate antiseptic solution in preoperative bathing has not been demonstrated when compared to placebo, non-medicated soaps, or the absence of bathing ( 4 , 10 ). Therefore, full-body washing with a soap-based solution—whether or not it contains an antimicrobial agent or antiseptic—is considered good practice and is widely encouraged in the literature. It is recommended at least on the night before surgery. Studies have yet to determine the precise benefits, the optimal time interval between bathing and surgery, or the ideal number of preoperative baths ( 1 ).

**Hair Removal:** There is no formal recommendation regarding hair removal at the surgical incision site, despite its routine use in clinical practice ( 11 ). A meta-analysis by the Cochrane Collaboration found no significant difference in SSI risk between no hair removal, chemical depilation, and clipping ( 12 ). However, shaving with a razor was associated with a higher risk of infection ( 13 ). Another meta-analysis found no significant differences between shaving, clipping, no hair removal and depilatory cream in the frequency of surgical site infections ( 14 ). Regarding the timing of hair removal, no difference in infection risk was observed whether it was performed the day before or on the day of surgery ( 12 ).

**Antisepsis:** The risk of SSI in clean surgeries ranges from 0.6% to 5%. The primary source of contamination in clean procedures is the patient's skin microbiota. There are no comparative studies evaluating the effectiveness of performing antisepsis versus omitting it. A meta-analysis comparing alcohol-based solutions with aqueous solutions of chlorhexidine and iodine-based agents demonstrated superior performance for alcohol-based solutions ( 1 ). Among alcohol-based solutions, 0.5% chlorhexidine was more effective than povidone-iodine, although this conclusion was based on a single study with a high risk of bias ( 15 ). No evidence supports the reapplication of the antiseptic solution at the end of the procedure or the use of antimicrobial sealants on the skin after intraoperative antisepsis ( 1 , 16 ). Systematic reviews and meta-analyses have found no benefit in using adhesive incise drapes, whether coated with povidone-iodine (PVPI) or not, compared to their omission ( 17 , 18 ).

**Surgical Site Irrigation:** There is no significant difference in SSI rates with or without irrigation of the incised surgical site ( 19 ). The use of iodophor-based aqueous solutions for peritoneal lavage is not necessary in cases where there is no local contamination or infection ( 1 ). The application of plastic adhesive wound drapes, with or without antimicrobial properties, over a primarily closed surgical wound does not reduce the risk of SSI ( 1 ).

**Body Temperature Regulation:** Perioperative warming and maintenance of normothermia are recommended to reduce the risk of surgical infections ( 1 , 20 , 21 ). However, there are no randomized studies establishing the lower acceptable limit of body temperature, the optimal strategy for thermal maintenance, or the ideal duration of normothermia.

**Recommendations:**

- Maintain preoperative blood glucose levels below 200 mg/dL; use insulin therapy when necessary. (GRADE: moderate, strong)
- Perform a full-body wash with a soap-based solution, with or without antiseptics, at least the night before surgery. (GRADE: low, strong)
- Hair removal is not mandatory. Clipping is the preferred method for hair removal at the surgical incision site. (GRADE: high, strong)
- Apply a 0.5% alcohol-based chlorhexidine solution for preoperative skin antisepsis. (GRADE: low, strong)
- There is no recommendation for irrigation of the incised surgical site or the abdominal cavity without contamination or infection. (GRADE: low, weak)
- The use of antibiotic-containing sealants or adhesive drapes (whether coated with povidone-iodine or not) following antisepsis, surgical site irrigation, or abdominal cavity irrigation in the absence of contamination or infection is not recommended. (GRADE: low, weak)
- Implement perioperative warming and maintain normothermia. (GRADE: moderate; strong)

#### Antibiotic Prophylaxis Measures for Surgical Infection Prevention

Perioperative antibiotic prophylaxis is administered to individuals without active infection, aiming to achieve serum and tissue drug concentrations above the minimum inhibitory concentration of potential infectious agents, thereby preventing the development of clinical infection ( 22 ). In urological surgeries, the involvement of potentially colonized structures or organs (e.g., the urinary tract or intestines), as well as the use of catheters and drains, increases the risk of infection due to contamination of the surgical site by endogenous microorganisms, bacterial dissemination from a possible septic focus, or contamination of sterile urine ( 22 - 24 ).

The decision to administer prophylactic antibiotics in urological procedures—whether open or endoscopic—should be individualized, weighing the risks and benefits of prophylaxis versus no prophylaxis ( 22 ).

Preoperative detection of bacteriuria aims to reduce infection risk and optimize the effectiveness of prophylactic antibiotic coverage. Urine culture is the preferred diagnostic method for detecting bacteriuria. Comparative studies with alternative diagnostic techniques, including some with faster turnaround times, have shown that none are suitable replacements for urine culture ( 25 ).

Patients with asymptomatic bacteriuria should only receive antibiotic treatment if they are undergoing procedures that involve breaching the urothelium ( 26 , 27 ). Antibiotics for prophylaxis can be administered via parenteral (most common), oral, or topical irrigation routes. The antibiotic must be administered before the surgical incision. The timing of parenteral administration should be guided by the drug's pharmacokinetic and pharmacodynamic properties to ensure adequate bactericidal serum and tissue concentrations during incision or at the start of a non-incisional procedure, with levels maintained throughout the surgery ( 28 ). Initially introduced in the CDC's 1999 guidelines, this recommendation was reaffirmed in an updated publication by the same regulatory body in 2017 ( 1 ). There are no randomized studies, systematic reviews, or meta-analyses directly comparing different timing intervals for prophylactic antibiotic administration. However, multiple international guidelines recommend administration within 60 minutes before incision for most antibiotics and 60 to 120 minutes prior for vancomycin and fluoroquinolones ( 1 , 18 ).

A retrospective study comparing no prophylaxis versus different antibiotics with different administration regimens demonstrated that antibiotic prophylaxis effectively reduces surgical site infections, favoring agents with a half-life exceeding four hours and showing that single-dose prophylaxis is as effective as a three-day regimen, with a lower risk of selecting resistant bacterial strains ( 22 ). Short half-life antibiotics may fail to maintain adequate serum levels for minimum bacterial inhibitory concentrations throughout surgery. However, there is limited evidence on the benefits and risks of intraoperative antibiotic re-administration. Based on expert opinion and guideline reviews, a repeat dose is recommended for prolonged surgeries (>3–4 hours or exceeding two half-lives of the antibiotic) or in blood loss greater than 1,500 mL ( 1 , 29 ). A recent meta-analysis found no difference in infection risk between single-dose and multiple-dose prophylactic antibiotic regimens in orthopedic surgeries involving implants ( 30 ). No randomized studies have evaluated this comparison in urological procedures.

There are no controlled studies supporting the need for antibiotic dose adjustments based on patient weight or body mass index. Current recommendations for weight-based dose adjustments are based on guideline reviews and expert opinion ( 1 ).

After skin closure or completion of an endoscopic procedure, there is no indication for additional antibiotic doses, even if surgical drainage is maintained for clean or potentially contaminated surgeries. This recommendation is supported by randomized studies across multiple surgical specialties, including cardiac, thoracic, vascular, orthopedic, head and neck, gynecological, and gastrointestinal procedures ( 1 ).

**Recommendations:**

- Asymptomatic bacteriuria should be treated (based on antibiotic susceptibility testing) ONLY for diagnostic or surgical procedures that breach the mucosal barrier of the urinary tract. Urine culture is the method of choice for diagnosing bacteriuria. (GRADE moderate, strong)
- Parenteral antibiotic administration should be timed to ensure adequate tissue concentration before surgical incision or endoscopic intervention. For most antibiotics, the recommended administration window is within 60 minutes before incision and between 60 and 120 minutes for vancomycin and fluoroquinolones. (GRADE low, strong)
- A repeat antibiotic dose should be administered in procedures exceeding two antibiotic half-lives or when blood loss exceeds 1,500 mL. (GRADE low, strong)
- There is no recommendation for antibiotic dose adjustment based on body weight in adults. (GRADE very low, weak)
- No additional antibiotic doses should be administered after skin closure or completion of an endoscopic procedure, even if surgical drainage is maintained for clean or potentially contaminated surgeries. (GRADE moderate, strong)

### Specific Measures for Surgical Infection Prophylaxis in Urological Surgeries

#### Urodynamic Study

Urinary tract infection (UTI) is the most common complication of urodynamic studies, although its exact incidence remains unclear due to variations in definitions across studies ( 31 - 36 ). While antibiotic prophylaxis has been shown to reduce the incidence of asymptomatic bacteriuria, its clinical impact and the cost-effectiveness of antimicrobial prophylaxis in preventing UTIs are not fully established ( 37 - 40 ). As a result, most scientific society guidelines recommend reserving antibiotic prophylaxis for patients at higher risk of developing UTIs, including neurogenic lower urinary tract dysfunction due to suprasacral spinal cord injury, age over 70 years, asymptomatic bacteriuria, immunosuppression, use of urinary catheters (indwelling, cystostomy, or clean intermittent catheterization), total orthopedic prosthesis implantation with risk factors for local infection due to bacteremia (e.g., inflammatory arthritis, diabetes mellitus, HIV infection), and patients with post-void residual volume exceeding 50 mL. ( 31 , 33 , 41 - 54 )

Due to the globally high resistance rates of uropathogens to fluoroquinolones, we recommend avoiding this class of antibiotics for urodynamic prophylaxis ( 55 - 59 ). Single-dose antibiotic prophylaxis before the urodynamic study has been described using the following regimens: fosfomycin-trometamol (3g, 2 hours before the procedure), nitrofurantoin (100 mg, 2 hours before the procedure), sulfamethoxazole-trimethoprim (800 mg/160 mg, 1 hour before the procedure), or cephalexin (500mg, 1hr. before the procedure) ( 31 , 60 ).

Considering the latest recommendations for managing acute and recurrent cystitis, the susceptibility profile of uropathogens, and the efficacy of different agents against urinary tract microorganisms, fosfomycin-trometamol or nitrofurantoin should be the first-choice prophylactic regimen ( 61 - 63 ).

**Recommendations:**

- Administer antibiotic prophylaxis for patients undergoing urodynamic studies who have risk factors for UTI development. (GRADE moderate, strong)
- Avoid the use of fluoroquinolones. (GRADE high, strong)
- Use fosfomycin-trometamol (3g, 2 hrs. before the procedure) or nitrofurantoin (100mg, 2 hrs. before the procedure) as the first-choice prophylactic regimen. (GRADE low, weak)

#### Prostate Biopsy

Prostate biopsy is considered a high-risk, contaminated, invasive procedure and therefore requires antibiotic prophylaxis ( 64 ). Risk factors for post-biopsy infection include prior antibiotic use within the last six months, the presence of an indwelling urinary catheter, and a history of recurrent prostatitis ( 65 , 66 ).

There is evidence that non-antibiotic measures can effectively reduce infection-related complications. A meta-analysis of eight randomized studies including 1,786 patients evidenced a 50% reduction in infection rates with rectal cleansing using povidone-iodine ( 67 ).

The necessity and efficacy of antibiotic prophylaxis were demonstrated in a Cochrane Collaboration review of nine randomized studies with 3,599 patients, comparing those who received antibiotics to a control group prior to transrectal prostate biopsy ( 68 ). The study showed a 63% reduction in infectious events in the prophylaxis group: bacteriuria (14.8% vs. 3.9%), bacteremia (8.6% vs. 2.1%), fever (10.8% vs. 4.0%), urinary tract infection (9.0% vs. 3.3%), and hospitalization (3.3% vs. 0.3%). Another meta-analysis of 11 studies with 1,753 patients reported a 44% reduction in post-transrectal biopsy infection rates with antibiotic prophylaxis ( 69 ). Based on these findings, international consensus guidelines strongly recommend antibiotic prophylaxis for all patients undergoing a transrectal prostate biopsy, with a high level of evidence and a strong grade of recommendation ( 65 , 66 , 70 ).

Although there is a consensus that antibiotic prophylaxis is necessary, there is significant variability in clinical practice regarding the choice of antibiotics ( 64 ). The selected antibiotic should target gram-negative bacteria ( *Enterobacteriaceae* ), achieve good penetration into prostate tissue, and reach the minimum inhibitory concentration at the time of the procedure. It is also essential to consider local resistance patterns of *Enterobacteriaceae* to commonly used antibiotics. The most frequently recommended antibiotics are fluoroquinolones and cephalosporins ( 66 , 71 ).

Although most studies have utilized fluoroquinolones, there has been a progressive global increase in Enterobacteriaceae resistance to this class of antibiotics, with reported resistance rates ranging from 18% to 83% in patients undergoing prostate biopsy. For instance, there has been a rise in post-biopsy infectious complications from 1% in 1996 to 4.1% in 2002 in Canada ( 68 , 69 ). A prospective study conducted in São Paulo, Brazil, analyzing 623 transrectal biopsies, reported an infectious complication rate of 6.4%, with 92% of infections caused by *E. coli* , of which 94% were fluoroquinolone-resistant ( 72 ). In light of these findings, the American Urological Association (AUA) recommends avoiding fluoroquinolone use in regions where resistance rates exceed 20% ( 66 ). Similarly, the European Medicines Agency and the European Association of Urology (EAU) contraindicate fluoroquinolones for transrectal biopsy prophylaxis, not only due to increasing resistance rates but also because of their musculoskeletal side effects ( 65 , 73 , 74 ).

A systematic review found similar infection complication rates between fluoroquinolones, cephalosporins, and aminoglycosides, while fosfomycin significantly reduced infection complications compared to fluoroquinolones ( 69 ). A review and meta-analysis of five studies—three randomized and two retrospective—comparing 1,447 patients who received fosfomycin versus fluoroquinolones showed a significant 80% reduction in post-biopsy infection rates with fosfomycin, therefore confirming fosfomycin's role as an alternative ( 75 ).

Extended prophylaxis refers to the use of two antibiotics. Although this approach contradicts the principle of using a single agent for prophylaxis, its rationale lies in providing broader coverage against potential resistance to a single antibiotic class ( 73 ). A meta-analysis of three randomized studies demonstrated that adding an aminoglycoside to a fluoroquinolone or cephalosporin reduced the risk of infectious complications by 65% ( 71 ). Similarly, a review of nine studies involving 2,597 patients confirmed extended prophylaxis's superiority ( 69 ).

A meta-analysis involving 12,320 individuals evaluated the use of rectal swab cultures to guide targeted antibiotic prophylaxis. The analysis demonstrated that targeted prophylaxis significantly reduced infection rates from 3.4% to 0.8% compared to empirical antibiotic therapy. However, only one of the nine included studies was randomized, with the remaining being retrospective series ( 76 ). Another meta-analysis of six studies involving 1,511 prostate biopsies found that empirical fluoroquinolone therapy was inferior to targeted antibiotic prophylaxis ( 69 ).

A systematic review, along with European and American consensus guidelines on transrectal biopsy prophylaxis, concluded that in regions with high fluoroquinolone resistance rates, extended prophylaxis, targeted prophylaxis, or the use of alternative antibiotics such as fosfomycin is recommended ( 66 , 69 , 77 ). The lowest effective dose and shortest duration should be used to minimize antibiotic-related side effects and reduce the risk of resistance. There is ongoing controversy regarding the optimal duration of prophylaxis and the timing of its initiation for transrectal biopsy.

There is no consensus or standardized regimen for antibiotic prophylaxis in transrectal biopsy. However, a meta-analysis found that single-dose prophylaxis was inferior to a one-day regimen ( 69 ). A Cochrane review comparing one-day versus three-day prophylaxis reported similar infection rates, fever, and hospitalization but a lower incidence of bacteriuria in the three-day prophylaxis group ( 68 ). A meta-analysis of 17 studies including 3,999 patients found that short-term prophylaxis (ranging from a single dose to three days) was associated with a higher incidence of infectious complications than long-term prophylaxis (one to seven days) using fluoroquinolones. The study also evidenced that the administration route and the antibiotic initiation timing (ranging from 12 to 2 hours before biopsy) did not influence infection rates ( 69 ). Another systematic review and meta-analysis, including 13 randomized studies with 3,389 patients, compared longer prophylaxis durations (three, five, and seven days) to a one-day regimen and found a 39% lower infection rate in favor of more extended prophylaxis ( 71 ). Subgroup analyses showed that while three-day prophylaxis was superior to one day, extending prophylaxis to five or seven days did not provide additional benefits ( 71 ).

In settings with high fluoroquinolone resistance rates, alternative prophylactic regimens are recommended. These include fosfomycin (3g orally, 3 hours before biopsy, followed by a second 3g dose 24 hours after the procedure); aminoglycosides such as amikacin (15 mg/kg IV) or gentamicin (3 mg/kg IV), administered 30 to 60 minutes prior to biopsy; or ceftriaxone (1g IV, 30 minutes before biopsy) followed by a second-generation cephalosporin for three days ( 69 ).

The most effective strategy for preventing infection after prostate biopsy is transitioning to the transperineal approach. A meta-analysis of 11 studies comparing 1,644 transperineal and 1,634 transrectal biopsies found a 74% lower infection rate with the transperineal route ( 77 ). A systematic review including 165 studies and 162,577 patients reported sepsis rates of 0.1% for transperineal biopsies and 0.9% for transrectal biopsies ( 78 ).

The European Association of Urology (EAU) recently updated its meta-analysis, now including 11 studies with 3,131 patients comparing infectious complications following transperineal versus transrectal prostate biopsy. Incorporating randomized trials published in 2024, the analysis concluded that the transperineal approach is significantly associated with lower rates of infectious complications (p = 0.03) ( 79 ).

Transperineal prostate biopsy is considered a clean procedure, as it avoids needle passage through the rectum and thus requires only antibiotic coverage for skin flora of the perineal region ( 65 , 70 , 73 , 74 ). There are no randomized studies evaluating different antibiotic regimens for transperineal biopsy prophylaxis. Most available data come from single-center case series, which commonly used first- or second-generation cephalosporins, such as cefazolin or cefuroxime, with reported infectious complication rates below 1% ( 65 , 80 - 83 ).

Two recently published meta-analyses evaluated the feasibility of performing transperineal prostate biopsy without antibiotic prophylaxis. Basourakos et al. reviewed 106 studies comparing 37,805 patients who received prophylaxis to 4,772 who did not and found no significant difference in sepsis rates between the groups (0.05% vs. 0.08%, p = 0.2). However, when considering minor complications, antibiotics had a significant advantage (0.55% vs. 1.22%, p < 0.01) ( 80 ). Another review of eight studies—four retrospective and four prospective non-randomized—compared 3,662 transperineal biopsies performed with or without antibiotic prophylaxis. This analysis found no significant difference in the incidence of fever (0.69% vs. 0.47%) or urinary tract infection (0.13% vs. 0.31%) ( 81 ). A key limitation of this review was the moderate to high risk of bias in seven of the included studies. Supporting the potential for transperineal biopsy without antibiotics, Hu et al. published a multicenter randomized trial in 2024, in which 742 patients were assigned to undergo either transperineal biopsy without antibiotics or transrectal biopsy with standard antibiotic prophylaxis. The study found no infectious complications in the transperineal group, compared to a 1.6% infection rate in the transrectal group (p = 0.015) ( 84 ).

Consensus guidelines still recommend administering a single intravenous dose of 1 gram of cefazolin immediately before transperineal biopsy to provide coverage against skin flora. Additionally, perineal skin antisepsis with povidone-iodine or chlorhexidine is advised ( 65 , 80 , 81 ). For patients with beta-lactam allergy, oral sulfamethoxazole-trimethoprim is recommended as an alternative prophylactic option ( 85 ).

**Recommendations:**

- Rectal cleansing with povidone-iodine immediately before transrectal biopsy is recommended. (GRADE high, strong)
- Antibiotic prophylaxis for transrectal prostate biopsy should be administered for 1 to 3 days, starting between 12 and 2 hours before the procedure. (GRADE high, strong)
- Fluoroquinolones (only if local bacterial resistance is <20%) and cephalosporins are recommended for transrectal biopsy prophylaxis. (GRADE high, strong)
- Fosfomycin, cephalosporins, and aminoglycosides should be considered alternatives to fluoroquinolones. (GRADE high, strong)
- In place of fluoroquinolones, extended prophylaxis with the addition of a broad-spectrum parenteral antibiotic (GRADE moderate, strong) or targeted antibiotic therapy based on rectal swab cultures (GRADE high, strong) is recommended.
- Perineal skin antisepsis with povidone-iodine or chlorhexidine is recommended before biopsy. (GRADE low, strong)
- For transperineal prostate biopsy, single-dose antibiotic prophylaxis with intravenous cefazolin is recommended. For patients with beta-lactam allergy, single-dose oral sulfamethoxazole may be used as an alternative. (GRADE low, weak)

#### Urethrocystoscopy

Antibiotic prophylaxis during urethrocystoscopy has been shown to reduce the risk of urinary tract infection (UTI) but does not lower the incidence of UTIs with systemic manifestations ( 41 , 86 , 87 ). However, existing studies are of low methodological quality, which limits the ability to support a recommendation for routine prophylaxis in all patients. Therefore, antibiotic prophylaxis is not recommended in asymptomatic patients with sterile urine cultures ( 77 , 64 ). In contrast, antibiotic prophylaxis is indicated for patients with risk factors for developing bacteremia, such as immunosuppression, the presence of an indwelling urinary catheter, heavy smoking history, malnutrition, prolonged hospitalization, pregnancy with asymptomatic bacteriuria, diabetes mellitus, neurogenic lower urinary tract dysfunction due to suprasacral spinal cord injury, and advanced age. Antibiotic prophylaxis should also be considered in cases where extensive mucosal trauma is expected during the procedure ( 64 ).

Due to the global rise in bacterial resistance of uropathogenic enterobacteria to fluoroquinolones, this class of antibiotics should be avoided for prophylaxis in urethrocystoscopy ( 55 - 58 , 88 , 89 ). As alternatives, several prophylactic regimens have been described and may be administered before or after the procedure. These include amoxicillin combined with potassium clavulanate (500 mg + 125 mg, single dose, 2 hours before the procedure), cephalexin (500 mg, single dose, 2 hours before the procedure), fosfomycin trometamol (3 g, 3 hours before the procedure, with a second 3 g dose 24 hours later), and nitrofurantoin (50 mg immediately after the procedure, then every 6 hours for 2 days). These regimens are associated with mild and infrequent adverse effects ( 64 , 90 , 91 ).

**Recommendations**

- Antibiotic prophylaxis is not recommended for asymptomatic patients with a negative urine culture undergoing urethrocystoscopy. (GRADE moderate, strong)
- Antibiotic prophylaxis is recommended for patients undergoing urethrocystoscopy with risk factors for developing bacteremia. (GRADE moderate, strong)
- Fluoroquinolones are not recommended for antibiotic prophylaxis in urethrocystoscopy. (GRADE high, strong)

#### Extracorporeal Shock Wave Lithotripsy (ESWL) for Urinary Stones

Regarding antibiotic prophylaxis in patients undergoing ESWL, two meta-analyses—Lu et al. (2012) and Mrkobrada et al. (2015)—evaluated nine and eight randomized clinical trials, respectively, including data from 1,364 and 940 patients. Neither study demonstrated the benefit of prophylactic antibiotic use in preventing fever, bacteriuria, or any infectious outcomes following ESWL ( 92 , 93 , 70 ). During the same period, although with lower evidence strength, an ecological study based on a New Zealand database including over 10,000 cases also found no significant benefit from the routine use of prophylactic antibiotics to prevent urinary tract infections after lithotripsy ( 94 ). More recently, a meta-analysis evaluating 16 randomized clinical trials and including 2,442 patients—incorporating several newer trials with stronger methodological quality—showed no benefit of antibiotic prophylaxis in preventing UTIs, even in patients with prior urinary tract manipulation ( 95 ).

**Recommendation**

Antibiotic prophylaxis is not recommended for patients without bacteriuria undergoing ESWL. (GRADE high, strong)

#### Endoscopic Ureterolithotripsy

A recent meta-analysis including 11 randomized clinical trials and 4,591 patients found that preoperative antibiotic prophylaxis did not reduce the risk of febrile urinary tract infection after endoscopic ureterolithotripsy. However, a single dose of antibiotic was effective in reducing pyuria and bacteriuria. The authors suggested that a single oral dose of prophylactic antibiotic was preferable due to its cost-effectiveness. These findings support the notion that, while prophylaxis may reduce subclinical infection, its clinical impact in preventing serious complications such as sepsis remains uncertain ( 96 ). Another study evaluating the incidence of systemic inflammatory response syndrome (SIRS) in patients receiving different types of antibiotics found no significant difference in serious complication rates between those who received no prophylaxis, a single dose, or two doses. Subgroup analysis, however, indicated a higher likelihood of SIRS in patients with stone burdens larger than 200 mm² who did not receive antibiotic prophylaxis ( 97 ). A cohort study compared extended antibiotic prophylaxis to standard single-dose prophylaxis in patients undergoing flexible ureteroscopy with negative urine cultures. Those who received extended prophylaxis had twice the risk of developing cystitis and isolating multidrug-resistant organisms, with no difference in the rates of pyelonephritis or urosepsis. These findings reinforce the recommendation for single-dose preoperative prophylaxis ( 98 ).

The adoption of personalized prophylactic strategies—such as the use of preoperative urine cultures, selection of antibiotics based on local bacterial resistance profiles, and the application of technologies like next-generation sequencing (NGS) to guide antibiotic choice—has been shown to reduce the incidence of clinically significant UTIs ( 99 ).

**Recommendation**

- Single-dose antibiotic prophylaxis should be administered preoperatively for endoscopic ureterolithotripsy, guided by local antibiotic resistance patterns. (GRADE high, strong)

#### Percutaneous Nephrolithotomy

A meta-analysis including 1,549 patients from 13 randomized clinical trials demonstrated that preoperative antibiotic prophylaxis in PCNL significantly reduces postoperative rates of sepsis and fever. Prolonged use of antibiotics after the procedure showed no additional benefit in preventing sepsis and was associated with an increased incidence of fever ( 100 ). Another meta-analysis, which included seven randomized clinical trials with 649 patients, found no benefit to extended antibiotic prophylaxis—whether postoperatively or continued until nephrostomy tube removal—further reinforcing the concept that prophylaxis should be limited to the anesthetic induction period ( 101 ).

In high-risk patients—such as those with staghorn calculi, indwelling catheters or stents, or known preoperative colonization—a meta-analysis of 10 randomized clinical trials found that prolonged antibiotic regimens may also be beneficial while single-dose prophylaxis is effective ( 102 ). Another meta-analysis, which included five studies (three randomized clinical trials and two observational studies), evaluated extended preoperative prophylaxis and found that combining a one-week preoperative antibiotic course plus a dose of intravenous antibiotics at the time of surgery reduced the risk of infection and sepsis in high-risk patients. This regimen also lowered the rates of positive intraoperative urine and stone cultures without increasing the incidence of postoperative fever ( 103 ). Comparisons between different antibiotic regimens did not reveal significant differences in infection rates associated with the procedure ( 104 ).

**Recommendations**

- Single-dose antibiotic prophylaxis should be administered preoperatively in patients undergoing PCNL. (GRADE high, strong)
- In high-risk patients, extended preoperative antibiotic prophylaxis (7 days) and one dose of intravenous antibiotics at the time of surgery is recommended. (GRADE high, strong)

#### Transurethral Resection of the Prostate (TURP) and Prostatic Enucleation

A meta-analysis by Dahm et al., initially published in 2009 and updated in 2017, evaluated 39 randomized clinical trials and demonstrated that antibiotic prophylaxis in TURP significantly reduces the risk of postoperative infections. Specifically, it showed a 50% relative risk reduction for sepsis (95% CI: 27–96%), a 64% reduction in febrile complications (95% CI: 55–75%), and a 37% reduction in bacteriuria (95% CI: 32–41%) ( 105 ). Regarding the duration of antibiotic use, patients with sterile urine cultures in the preoperative period showed similar infection rates regardless of whether they received a single dose or extended antibiotic regimens when undergoing prostatic enucleation ( 106 ). More recently, Baten et al. observed that patients without preoperative catheters or pyuria had low rates of febrile infectious complications, suggesting that antibiotic prophylaxis may not be necessary in such cases ( 107 ).

**Recommendations**

- Preoperative antibiotic prophylaxis is recommended for men without bacteriuria undergoing TURP or enucleation of the prostate. (GRADE high, strong)

#### Transurethral Resection of Bladder Tumor (TURBT)

A meta-analysis by Bausch et al., which included seven studies and 1,725 participants, did not demonstrate significant benefits from antibiotic prophylaxis in patients undergoing bladder tumor resection. The incidence of symptomatic bacteriuria (OR = 1.55 [0.73–3.31], 95% CI) and asymptomatic bacteriuria (OR = 0.43 [0.18–1.04], 95% CI) did not differ significantly between the groups analyzed ( 108 ).

However, antibiotic prophylaxis was found to be beneficial in specific high-risk populations, including patients with a history of pelvic radiotherapy, advanced age, prolonged preoperative hospital stay, tumors larger than 2 cm, and those with preoperative pyuria or bacteriuria ( 109 , 110 ).

A randomized clinical trial evaluating high-risk patients demonstrated the non-inferiority of oral fosfomycin administered the day before the procedure compared to intravenous cefoxitin given perioperatively ( 111 ).

**Recommendation**

- Antibiotic prophylaxis is recommended for high-risk patients undergoing TURBT. (GRADE high, strong)

#### Intravesical Botulinum Toxin Injection

No randomized clinical trials are available to guide antibiotic prophylaxis for intravesical botulinum toxin injection. Current evidence suggests a trend toward omitting prophylactic antibiotics, even in asymptomatic bacteriuria ( 112 - 115 ). An important exception involves patients undergoing botulinum toxin treatment for neurogenic lower urinary tract dysfunction secondary to suprasacral spinal cord injury ( 113 - 115 ). In this population, antibiotic prophylaxis is recommended on the day of the procedure, with continuation for 1 to 3 days following the injection ( 115 ). A retrospective study conducted in a Brazilian cohort demonstrated that a single antibiotic dose at the time of anesthesia induction was associated with a UTI rate of only 1.8%, supporting the efficacy of this approach ( 113 ).

Among the available antibiotics, cefazolin is recommended as the first-line option, with ciprofloxacin reserved for exceptional cases ( 113 ). Although there is currently no clinical data supporting the prophylactic use of fosfomycin trometamol in this specific patient group, a recent epidemiological study showed that urinary pathogens isolated from neurologic patients undergoing intravesical botulinum toxin injections generally do not exhibit resistance to fosfomycin, in contrast to the resistance patterns seen with other antibiotic classes ( 116 ).

**Recommendations**

- Antibiotic prophylaxis is recommended for patients with asymptomatic bacteriuria and suprasacral spinal cord injury undergoing intravesical botulinum toxin injection. (GRADE moderate, strong)
- When antibiotic prophylaxis is indicated, a single intravenous dose of cefazolin during anesthesia induction is recommended as the first-line option for intravesical botulinum toxin procedures. (GRADE moderate, strong)

#### Surgical Correction of Female Stress Urinary Incontinence

In general, there is a growing tendency not to recommend routine antibiotic prophylaxis for surgical correction of female stress urinary incontinence, primarily due to the low postoperative infection rates associated with current surgical techniques ( 117 - 120 ). A retrospective controlled study from Norway involving more than 9,000 patients found a reduction in infections with antibiotic prophylaxis; however, the overall infection rate was only 1.2% ( 117 ). Supporting these findings, a case series of 174 patients who did not receive prophylaxis reported no surgical site infections and only an 8% rate of asymptomatic bacteriuria ( 119 ). The only available double-blind, randomized controlled trial on the topic, which compared a single 1 g dose of intravenous cefazolin administered at anesthesia induction to a placebo, was terminated early due to the very low infection rates observed ( 120 ).

**Recommendation**

- Antibiotic prophylaxis is not recommended for mid-urethral sling surgeries in women. (GRADE moderate, strong)

#### Surgical Correction of Pelvic Organ Prolapse

In general, antibiotic prophylaxis is recommended for surgical correction of pelvic organ prolapse ( 64 , 121 - 123 ). For procedures performed via the vaginal or abdominal route without the use of synthetic mesh, postoperative infection rates are reduced with a single intravenous dose of cefazolin 2 g (or 3 g for patients over 120 kg), administered one hour before the surgical incision ( 64 , 121 , 124 ).

In cases involving synthetic mesh placement, there is no standardized protocol; however, better efficacy has been observed using at least two combined intravenous antibiotics, administered one hour before the incision and continued for the first 24 hours postoperatively. Commonly used combinations include clindamycin and gentamicin, aminoglycoside and vancomycin, aztreonam and vancomycin, aminoglycoside and a second-generation cephalosporin, or aztreonam and a second-generation cephalosporin ( 64 , 122 ).

**Recommendations**

- For pelvic organ prolapse repair surgeries without synthetic mesh, cefazolin is recommended as the first-line agent for antibiotic prophylaxis. (GRADE moderate, strong)
- For pelvic organ prolapse repair surgeries with synthetic mesh, combined antibiotic prophylaxis is recommended. (GRADE moderate, strong)

#### Urologic Prosthesis Implantation

To date, no randomized placebo-controlled trials have directly compared preoperative antibiotic administration with no prophylaxis in urologic prosthesis implantation. Nevertheless, the benefits of antibiotic prophylaxis in this setting are widely accepted based on evidence from other surgical fields. Studies in closed fracture repair and inguinal hernia repair with mesh have shown that appropriate prophylaxis with broad-spectrum, long-acting antibiotics significantly reduces the incidence of surgical site infections and early postoperative complications ( 125 - 127 ). For skin incisions where prosthetic devices are to be implanted, as well as for groin and perineal incisions—where the risk of surgical site infection may be higher—recent guidelines recommend preoperative antibiotic prophylaxis ( 64 , 128 - 137 ). In surgeries involving prosthesis implantation, the use of meticulous surgical techniques combined with appropriate antibiotic prophylaxis against both Gram-positive and Gram-negative bacteria has been shown to reduce infection rates ( 129 ). A recommended prophylactic regimens consists of aminoglycosides (or aztreonam in cases of renal insufficiency) combined with a first- or second-generation cephalosporin or vancomycin. This combination is aimed at covering skin flora, particularly coagulase-negative staphylococci and Gram-negative bacilli, including *Pseudomonas* spp ( 64 , 128 ). Identifying and decolonizing patients colonized with nasal *Staphylococcus aureus* using mupirocin and chlorhexidine prior to surgery has been shown to reduce surgical site infection rates from 4.4% to 0.9% ( 131 ). Although recent studies have demonstrated a general decline in prosthetic infection rates, relatively high rates of methicillin-resistant *Staphylococcus* aureus (MRSA) and fungal infections have been increasingly reported in cultures from explanted infected penile prostheses ( 136 ). Regarding antifungal prophylaxis in prosthetic surgery, a recent retrospective study showed it reduced the risk of infection, and a recent systematic review has also highlighted the likely importance of antifungal prophylaxis ( 134 , 135 ). Nonetheless, further comparative studies are needed to justify the routine inclusion of antifungal agents in prophylactic protocols.

An analysis of explanted prostheses identified a wide range of pathogens, including Gram-positive and Gram-negative bacteria, *Candida* species, anaerobic bacteria, *Staphylococcus aureus* , and polymicrobial infections. These findings suggest that, even when following current antibiotic recommendations aimed at covering the majority of skin flora, the microorganisms isolated in this study were not adequately covered by AUA guideline-recommended regimens in 14% to 38% of cases ( 136 ).

Contemporary literature has also shown a reduction in penile prosthesis infection rates of approximately 50%, mainly attributed to the introduction of antibiotic-impregnated devices and antibiotic-coated implants, therefore, great importance is given to the topical solution to be used in prostheses ( 139 - 142 ). The most commonly used devices among penile prosthesis and artificial urinary sphincter incorporate various technologies for antibiotic delivery—either through pre-impregnation with antibiotics or surface coatings that allow the surgeon to soak the device in an antibiotic solution of choice prior to implantation, enhancing localized antimicrobial activity ( 143 - 145 ). In a large-cohort, multicentre, retrospective study of men with diabetes who received a Coloplast Titan™ implant, different solutions for implant dipping were evaluated, and the combination of vancomycin and gentamicin proved to be the most effective in preventing postoperative infection, explantation, and revision ( 143 ). In contrast, for artificial urinary sphincters, the presence of InhibiZone coating has not demonstrated a significant impact on infection rates or explantation risk, according to findings from a retrospective cohort study ( 144 ).

Although there are no systematic reviews or prospective studies supporting the effectiveness of antibiotic prophylaxis specifically for artificial urinary sphincters, AUA guidelines recommend a similar approach to that used for penile prostheses. Prophylaxis should consist of an aminoglycoside combined with either a first- or second-generation cephalosporin, or vancomycin ( 64 , 144 ).

For testicular prostheses, a review has suggested the use of pre-incision antibiotics, including vancomycin and gentamicin, although the level of evidence supporting this recommendation is low ( 64 , 132 , 146 ).

Regarding the postoperative extension of antibiotic prophylaxis, a survey of prosthetic surgeons in the United States revealed that 90% of respondents routinely prescribed postoperative oral antibiotics, with an average duration of 7 days. This practice prompted the publication of a consensus in which all panel experts reported prescribing oral antibiotics postoperatively for 5 to 14 days despite well-documented evidence in surgical literature highlighting the risks associated with prolonged postoperative antibiotic use ( 144 , 147 ). A 2020 consensus statement recommended that postoperative antibiotic use should be discontinued within 24 hours unless specific antimicrobial prophylactic regimens are justified for particular patient populations. In such cases, postoperative prescribing is often based on the individual surgeon's clinical judgment and aligned with local institutional guidelines ( 124 ). Current evidence suggests that routine postoperative antibiotic administration is unlikely to benefit patients undergoing implant surgery, and more randomized controlled trials are needed to inform stronger recommendations ( 148 ).

**Recommendations**

- Intravenous antibiotic prophylaxis is recommended for urologic prosthesis implantation. (GRADE low, strong)
- The recommended antibiotic regimen includes an aminoglycoside combined with a first- or second-generation cephalosporin, or vancomycin. (GRADE low, strong)
- There is currently no recommendation to include antifungals or to broaden the spectrum of antibiotic coverage beyond standard prophylaxis. (GRADE low, weak)
- Antibiotic irrigation of prostheses is recommended, particularly for devices with hydrophilic coatings, using gentamicin alone or combined with rifampin or vancomycin. (GRADE low, weak)
- No current evidence supports the routine use of postoperative antibiotics. (GRADE low, strong)

#### Open, Laparoscopic, and Robotic Urologic Surgery

Antibiotic prophylaxis in abdominal or pelvic urologic surgeries should guided by the patient's clinical condition, the type of procedure, and the expected microbial flora encountered during surgery ( 1 ). The classification of the surgical wound, based on its contamination risk, in combination with the local antimicrobial resistance profile and the nature of the intervention, should inform the choice of prophylactic antibiotics ( 1 , 149 ). The duration of antibiotic prophylaxis should not exceed 24 hours, with most procedures requiring only a single parenteral dose ( 64 , 150 ). There are no differences in prophylactic antibiotic regimens between open, laparoscopic, or robot-assisted techniques. All approaches follow the same principles with respect to antibiotic type, dosing, and duration.

#### Classification Based on Surgical Wound Type

Surgical wounds are classified into four categories, adapted for urologic procedures, and used to determine the need and type of prophylaxis ( 64 , 151 ).

#### Class I/Clean Procedures

These are surgeries that do not involve entry into the gastrointestinal or genitourinary tracts. For low-risk clean procedures in patients without significant comorbidities and with expected operative times under three hours—such as vasectomy, circumcision, and varicocelectomy—antibiotic prophylaxis is not indicated ( 64 , 152 ). For clean procedures with higher infectious potential—such as adrenalectomy, lymphadenectomy (pelvic or retroperitoneal), hydrocelectomy, and inguinal or scrotal orchiectomy—antibiotic prophylaxis should be considered with coverage targeting skin flora ( 64 , 153 - 155 ). A single parenteral dose of cefazolin is preferred, with clindamycin as an alternative in cases of beta-lactam allergy. Procedures involving prosthetic implantation require broader prophylactic strategies, which are addressed in a separate section.

#### Class II/Clean-contaminated procedures

These involve controlled entry into the genitourinary tract, such as radical or partial nephrectomy, pyeloplasty, prostatectomy, and partial cystectomy ( 64 ). Antibiotic prophylaxis is recommended for all cases, targeting gram-negative rods and *Enterococcus* . The recommended regimen is a single intravenous dose of cefazolin or trimethoprim-sulfamethoxazole. Alternative regimens include ampicillin/sulbactam; an aminoglycoside plus metronidazole; aztreonam plus metronidazole; an aminoglycoside plus clindamycin; or aztreonam plus clindamycin.

#### Class III/Contaminated procedures

These include surgeries involving bowel segments (e.g., colonic urinary diversions) or those performed in the presence of infected stones, such as percutaneous nephrolithotomy for struvite calculi ( 64 ). Antibiotic prophylaxis is recommended for all contaminated procedures due to the high risk of SSI or even systemic infection. For colorectal diversions, preoperative mechanical bowel preparation combined with oral antibiotics is recommended as it is linked to lower complication rates ( 156 - 158 ). The recommended oral agents include neomycin plus metronidazole or erythromycin base ( 64 ). These should be used in addition to systemic antibiotics that provide both aerobic and anaerobic coverage. A single dose of a first-generation cephalosporin, combined with metronidazole for anaerobic coverage, is considered first-line parenteral prophylaxis for colorectal diversions or any surgery involving the large bowel ( 64 ). Carbapenems (eg, ertapenem) are also a first-line option and should be reserved for multi-drug resistant bacteria. Third or higher generations of cephalosporins are not routinely recommended due to their association with increased rates of multidrug-resistant bacterial infections ( 159 , 160 ). Alternative regimens may include ampicilin/sulbactam, pipercillin/tazobactam, or ticarcillin/clavulanate.

In contrast, using small bowel segments for diversion does not require bowel preparation ( 161 , 162 ). The upper gastrointestinal tract harbors a less dense and less diverse microbial flora compared to the colon. Therefore, for non-obstructed small bowel procedures—such as cystectomy with small bowel urinary diversion—a single dose of cefazolin is generally recommended for prophylaxis ( 64 , 163 ). Alternative regimens include a single dose of clindamycin combined with an aminoglycoside, a second-generation cephalosporin, or a combination of an aminopenicillin with a β-lactamase inhibitor and metronidazole. ( 64 ).

A randomized, single-center, non-blinded, non-inferiority trial compared 24-hour versus extended (>48 hours, median 8 days) perioperative antibiotic prophylaxis in patients undergoing cystectomy with urinary diversion ( 164 ). The study found no significant differences between the two groups in terms of surgical site infections, all-cause mortality, febrile urinary tract infections, or length of hospital stay—supporting the non-inferiority of the shorter regimen.

#### Class IV/Dirty procedures

Class IV, or dirty procedures, include surgeries involving open trauma, abscesses, or extensive debridement—such as in cases of Fournier's gangrene—where infection is already present at the time of the procedure. By definition, all Class IV procedures are considered infected ( 64 ). In these cases, intraoperative cultures should be obtained to guide therapy. Until culture results are available, empiric antibiotic therapy should be initiated or continued, tailored to the likely pathogens based on the surgical site and clinical context.

**Recommendations**

- Prophylactic antibiotic regimens should not differ between open, laparoscopic, and robot-assisted urologic surgeries, as all follow the same evidence-based principles regarding agent selection, dosing, and timing. (GRADE very low, strong)
- Antibiotic prophylaxis is not routinely recommended for Class I (clean) procedures, except in cases involving prosthetic implantation or where surgical site infection risk may be increased. In such cases, single-dose antibiotic prophylaxis targeting skin flora is recommended. (GRADE low, strong)
- Antibiotic prophylaxis is recommended for all Class II (clean-contaminated) procedures, with single-dose coverage targeting gram-negative bacilli and *Enterococcus* . (GRADE very low, strong)
- Antibiotic prophylaxis is recommended for all Class III (contaminated) procedures (GRADE low, strong)
- For colorectal diversions, combined mechanical bowel preparation and oral antibiotics should be used in conjunction with parenteral agents providing both aerobic and anaerobic coverage. (GRADE low, strong)
- In small bowel urinary diversions, where bowel preparation is not required, a single preoperative dose of cefazolin is recommended. (GRADE low, strong)
- Antibiotic prophylaxis duration should not exceed 24 hours for patients undergoing cystectomy with urinary diversion. (GRADE moderate, strong)
- For Class IV (dirty) procedures, such as abscess drainage or debridement in the setting of active infection, empiric broad-spectrum antibiotics should be initiated or continued based on the expected pathogens. Intraoperative cultures should be obtained to guide therapy. (GRADE low, strong)

## CONCLUSIONS

The standardization of replicable prophylactic protocols in diagnostic and therapeutic urological procedures is a key component for ensuring patient safety and effective care. However, both the use of non-antibiotic measures and the prescription of antimicrobials for prophylactic purposes are often based on outdated information, empirical practice, or commercial motivations, lacking the necessary scientific support. Notably, antibiotic prophylaxis in urological surgical procedures remains an area with a paucity of high-level evidence studies. The definition of prophylactic strategies must consider not only the characteristics of the procedure itself but also patient-related factors such as prior bacterial colonization, a history of recurrent urinary tract infections, catheter use, previous urological manipulation, urinary tract malformations, and the patient's overall clinical and immunological status. Additionally, aspects related to the underlying disease and potential functional interference with other organs must be integrated into the decision-making process. Thus, evidence-based and risk-adapted prophylaxis is essential for reducing infectious complications in the urological setting.
